# Amelioration of thyroid dysfunction by magnesium in experimental diabetes may also prevent diabetes-induced renal impairment

**DOI:** 10.1016/j.heliyon.2019.e01660

**Published:** 2019-05-08

**Authors:** A.O. Ige, R.N. Chidi, E.E. Egbeluya, R.O. Jubreel, B.O. Adele, E.O. Adewoye

**Affiliations:** Applied and Environmental Physiology Unit, Department of Physiology, University of Ibadan, Nigeria

**Keywords:** Physiology, Metabolism

## Abstract

**Background:**

Diabetes mellitus has been reported to cause thyroid dysfunction, which may also impair renal function. Magnesium has been reported to exert ameliorative effects in diabetes mellitus. This study investigated thyroid and renal functions in experimental type-2-diabetic Wistar rats.

**Methods:**

Experimental type-2-diabetes was induced using short duration high-fat (30%) diet feeding followed by single-dose streptozotocin (35 mg/kg i.p.). Fifty rats were randomly divided into five equal groups consisting of control, diabetes untreated, diabetes treated with either magnesium (250 mg/kg) or metformin (250 mg/kg) and diabetes treated with both metformin and magnesium simultaneously.

All treatments were carried out orally for 14days post-diabetes induction. Body weight and blood glucose was monitored using the tail tipping method before diabetes induction and thereafter on days 1,7,14 post-treatment respectively. Thereafter, blood samples were collected by cardiac puncture after light anesthesia into plain and EDTA sample bottles. Total protein, albumin, globulin (plasma) and insulin (serum) were assayed in all samples obtained. Thyroid stimulating hormone (TSH), triiodothyronine, thyroxine was also evaluated (n = 5/group) in serum while blood urea nitrogen (BUN), creatinine was assessed (n = 5/group) in plasma. Kidney homogenates were obtained per group and analyzed for renal superoxide dismutase (SOD), reduced glutathione (GSH) and lipid peroxidation (MDA). Kidney histology was also evaluated per group using both Haematoxylin and Eosin and periodic acid Schiff stains.

**Results:**

Body weight, blood glucose, insulin, renal MDA was increased in diabetic untreated compared to other groups. Reductions (P < 0.05) in TSH, triiodothynine, Renal SOD and GSH levels where observed in diabetic untreated compared to other groups. Renal histology in diabetic untreated showed glomerula sclerosis, fused messengial cells and either collapsed tubular lumen or lumen with eosinophilic renal cast. These pathologies where partially reversed in the other experimental groups.

**Conclusion:**

This study suggests that thyroid and renal impairment may be present in experimental type-2-diabetes. Treatment with oral magnesium may cause a partial restoration of thyroid function that may impede the development of renal dysfunction.

## 1 Introduction

Diabetes and thyroid disorders are common endocrine disorders that have been shown to mutually influence each other [1]. Studies have shown that thyroid hormones contribute to the regulation of carbohydrate metabolism and pancreatic function [2, 3]. Thyroid hormones have also been observed to directly control insulin secretion with hypothyroidism causing a reduction in glucose-induced insulin secretion by beta cells while the response of beta cells to glucose or catecholamine is increased in hyperthyroidism as a result of an increase in beta cell mass [4]. Furthermore, thyroid hormones have been reported to exert pre-renal and direct renal effects resulting in alterations in cardiac blood flow, glomerular filtration rate, tubular secretory and re-absorptive processes as well as renal tubular physiology [5]. Specifically, hypothyroidism is associated with increased creatinine and reduced GFR while hyperthyroidism results in increased GFR as well as increased renin–angiotensin–aldosterone activation [5]. Altered thyroid states have also been reported to occur in diabetes mellitus resulting in reductions in thyroid stimulating hormone in triiodothyronine levels [6]. It is thus likely that in diabetes mellitus, thyroid and renal functions need to be continually monitored so as to prevent metabolic anomalies that may arise from thyroid dysfunction and as well as prevent the development of chronic kidney disease (CKD).

Oral magnesium supplementation in diabetes has been reported to exert beneficial effects in both rat and human studies [7, 8, 9]. Hypomagnesaemia has been observed in most diabetics and this has been reported to increase the susceptibility of developing long-term complications of diabetes mellitus including thyroid dysfunction and CKD [10]. Furthermore hypomagnesaemia has been independently associated with thyroid dysfunction especially hypothyroidism, as magnesium is essential for iodine utilization by the thyroid gland and conversion of inactive thyroxine (T_4_) to active tri-iodotyronine (T_3_) [11]. Hypomagnesaemia has also been reported to be a novel predictor of renal disease [12]. Hence it is likely that hypomagnesaemia as observed in diabetes could cause hypothyroidism, which in turn may cause impaired renal function. This is however unsubstantiated; in addition whether oral magnesium supplementation in diabetes mellitus ameliorates diabetes induced thyroid and renal function is yet to be investigated. This study was thus designed to investigate thyroid and renal functions in experimental type 2 diabetic rats treated orally with magnesium, metformin (a standard type 2 anti-diabetic drug) as well as simultaneous treatments with both metformin and magnesium respectively.

## 2 Materials and methods

### 2.1 Animals, groupings and methodology

Fifty (50) male Wistar rats with an average weight of 128.9 ± 5.5 g were housed in well-ventilated cages, exposed to alternate light and dark cycles, maintained at 25–28 °C, low relative humidity, fed standard rat chow and allowed free access to drinking water in accordance with guidelines and protocol approved by the Animal Care and Use Research Ethics Committee (ACUREC) of the University of Ibadan, Nigeria (Approval no.: UIACUREC/17/0090) as well as guidelines given by the National Research Council, USA [13].

Experimental type-2-diabetes was induced using the method of Srinivansan et al, [14]; briefly experimental animals were maintained on 30% high fat diet (HFD) (maize 12.1%, soya 19.1%, groundnut cake 19.1%, wheat 5.8%, fish meal 9.6%, cal-cium and phosphorus 1.9%, lysine 0.38%, methionine 0.38%, pre-mix 0.76%, salt 0.76%, and lard 30%) feeding for 2weeks followed by a single intraperitoneal injection of streptozotocin (35 mg/kg). Fifty rats were randomly divided into five equal groups consisting of control, diabetes untreated, diabetes treated with either magnesium (as magnesium chloride) alone (250 mg/kg) [8, 9] or metformin (250 mg/kg) [15] and diabetes treated with both metformin and magnesium simultaneously. All treatments were carried out orally for 14 days post diabetes induction.

### 2.2 Measurements and biochemical assay

Body weight was assessed throughout the duration of the study using a laboratory scale while blood glucose was monitored using the tail tipping method before diabetes induction and thereafter on days 1, 7, 14 post-treatment. Blood glucose was analyzed using an Accu-Chek active glucometer (Roche, Germany) that used the glucose oxidase method as the basis for its analysis. At the end of the experiment, blood samples were collected by cardiac puncture after light di ethyl ether anesthesia into plain (3mls) and EDTA sample bottles (3.5mls). Serum was separated from the samples in the plain bottles and analyzed for insulin (Calbiotech, USA) while plasma was obtained from blood collected into EDTA-lined sample bottles and analyzed for total protein, albumin (Randox Laboratories, United Kingdom) in all samples collected. Globulin level was derived mathematically from the total protein and albumin levels obtained. Serum (n = 5/group) was further analyzed for thyroid stimulating hormone, thyroxine (T_4_) and triiodotyronine (T_3_) level using ELISA kits (Calbiotech,USA) while in plasma (n = 5/group), blood urea nitrogen (BUN) and creatinine was evaluated.

Kidney samples were also obtained from five (5) animals in each group, weighed and homogenized on ice in 1.15% KCl buffer (pH = 7.4). The kidney homogenates were centrifuged at 10,000 rpm for 15 min at 4 °C and the clear supernatant obtained was analyzed for lipid peroxidation [16], superoxide dismutase [17] and reduced glutathione levels [18]. Kidney samples were obtained from the remaining five (5) animals in each group and analysed for structural changes using haematoxylin and eosin (H and E) stains while tubular changes were evaluated using Periodic Acid Schiff (PAS) reaction techniques respectively.

### 2.3 Statistical analysis

Data were presented as mean ± standard error of mean. Statistical significance at P < 0.05 was established using one-way Analysis of Variance (ANOVA) and Newman Keuls' post-hoc test.

## 3 Results

### 3.1 Body weight (g) changes in control and experimental animals

Animals in the control had 22.69% increase in body weight (g) by day 14 compared to day 0 values (116.8 ± 4.48 vs. 143.3 ± 717) (Table 1). Values obtained in the diabetic untreated group, magnesium treated diabetic, metformin treated diabetic as well as the magnesium and metformin co-treatment diabetic groups had 35.28%, 26.11%, 35.07% and 37.37% increase in body weight compared to their respective day 0 values (Table 1).

**Table 1:** Body weight changes in normal and treated animals.

	DAY O (Before DM)	Day 1 (After DM)	Day 7	Day 14
Control	116.8 ± 4.48	132.8 ± 6.04	142.5 ± 6.15	143.3 ± 7.17
Diabetic Untreated	127 ± 4.50	156.4 ± 5.33*	161.6 ± 6.73*	171 ± 8.40*
Diab + Mg	148.6 ± 4.95	177.6 ± 2.80*	181.5 ± 5.29*	187.4 ± 6.22*
Diab + Met	122.6 ± 9.77	157 ± 8.14*	158.5 ± 8.37*	165.6 ± 8.40*
Diab + Mg + Met	129.5 ± 4.86	163.1 ± 4.35*	162.4 ± 4.05*	167.9 ± 4.84*

***Values expressed as mean ± SEM; P<0.05***^∗^ indicates values that are significantly different (p < 0.05) compared to control. Day 0 = values obtained before induction of experimental diabetes; Day 1 = 14 days after diabetes had been induced using high fat diet and single-dose streptozotocin (35 mg/kg i.p.). Day 7 = 7 days of treatment post diabetes induction. Day 14 = 14 days of treatment post diabetes induction.

### 3.2 Biochemical analysis in control and experimental animals

**Fig. 1:**
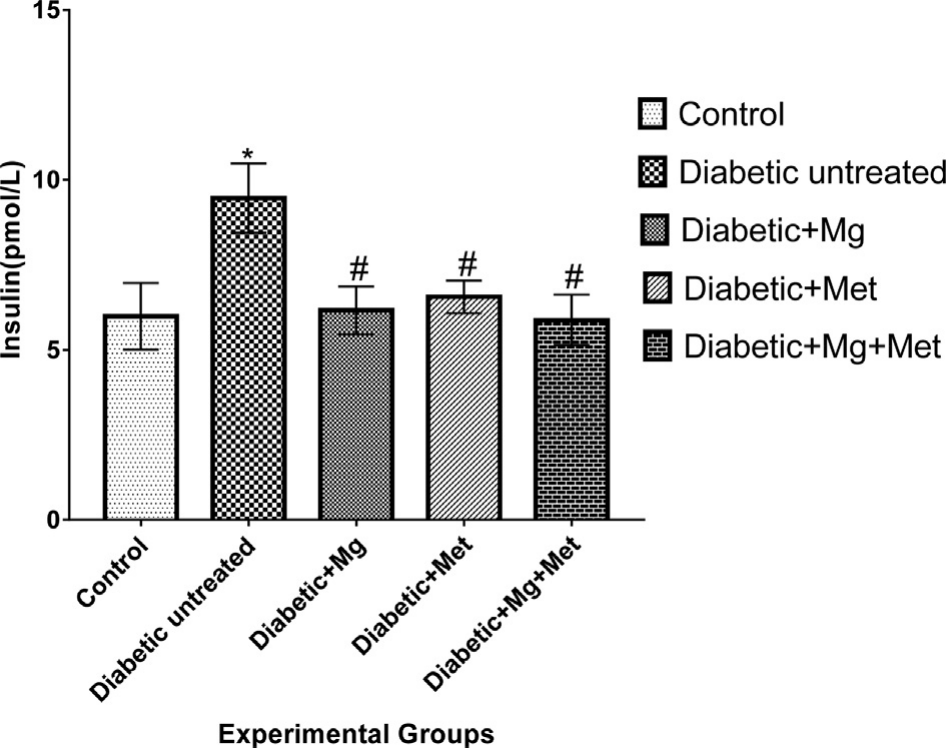
Serum Insulin Level in normal and treated rats. *Values expressed as mean ± SEM;*^∗^ indicates values that are significantly different (p < 0.05) compared to control. ^#^ indicates values that are significantly different (p < 0.05) compared to diabetic untreated group.

Animals in control group had an increase in blood glucose (mg/dl) at the end of the study (53.8 ± 2.13 vs. 80.0 ± 5.52) compared to initial values. However these values were still within the normal range. Animals in group 2 (diabetic untreated) had a significant increase in blood glucose level by day 1 (64.4 ± 3.54 vs. 389.3 ± 44.70) and this was sustained up to day 14 of the study (64.4 ± 3.54 vs. 368.0 ± 51.46) and significantly increased compared to control and all other experimental treatment groups (Table 2). Groups 3 (magnesium diabetic treated), 4 (metformin diabetic treated) and 5 (magnesium and metformin diabetic co-treated) had significantly increased (P < 0.05) blood glucose level on day 1 after diabetes induction compared to their respective day 0 values. On day 14, blood glucose values were significantly reduced (P < 0.05) compared to diabetic untreated animals (Table 2). Animals in the diabetic untreated group had insulin values that were significantly increased (P < 0.05) compared to control (36.7%), magnesium diabetic treated (34.9%), metformin treated (30.7%) and magnesium with metformin co-treatment (27.5%) group respectively (see Fig. 1). Increase in renal lipid peroxidation (0.73 ± 0.27 vs. 0.40 ± 0.07), reduced SOD activity (0.72 ± 0.03 vs. 1.33 ± 0.09) and a decline in reduced GSH level (40.49 ± 1.25 vs. 44.09 ± 2.00) in diabetic untreated group compared to control. Reduced glutathione was increased in the diabetic animals treated with magnesium (30.5%), metformin (21.6%) as well as magnesium and metformin co-treatment group (6.9%) compared to diabetic untreated group. Superoxide dismutase values obtained in the diabetic animals treated with magnesium, metformin as well as in the magnesium and metformin co-treatment group showed a 54.2%, 37.5% and 48.6% increase respectively compare to diabetic untreated group. Renal tissue lipid peroxidation was significantly reduced in the diabetic animals treated with magnesium (0.73 ± 0.27 vs. 0.22 ± 0.06), metformin (0.73 ± 0.27 vs. 0.42 ± 0.17), as well as in the magnesium and metformin co-treatment group (0.73 ± 0.27 vs. 0.41 ± 0.07) compared to diabetic untreated group (0.73 ± 0.27) (see Table 3).

**Table 2:** Blood glucose level of normal and treated animals.

	DAY O (Before DM)	Day 1 (After DM)	Day 7	Day 14
Control	53.8 ± 2.13	98.5 ± 5.48	81 ± 3.68	80 ± 5.52
Diabetic Untreated	64.4 ± 3.54	389.3 ± 44.70*	186.6 ± 31.34	368 ± 51.46
Diab + Mg	57.3 ± 2.69	353.8 ± 25.15*	115.8 ± 4.72*#	177 ± 11.42*#
Diab + Met	55.9 ± 1.80	382.7 ± 16.27*	96.1 ± 10.5*#	146 ± 3.16*#
Diab + Mg + Met	60.6 ± 3.02	359.13 ± 26.80*	98.2 ± 3.15*#	164.8 ± 16.60*#

***Values expressed as mean ± SEM; P<0.05***^∗^ indicates values that are significantly different (p < 0.05) compared to control. ^#^ indicates values that are significantly different (p < 0.05) compared to diabetic untreated group. Day 0 = values obtained before induction of experimental diabetes; Day 1 = 14 days after diabetes had been induced using high fat diet and single-dose streptozotocin (35 mg/kg i.p.). Day 7 = 7 days of treatment post diabetes induction. Day 14 = 14 days of treatment post diabetes induction.

**Table 4:** Thyroid function in normal and experimental animals.

	TSH	T4	T3
Control	1.12 ± 0.29	21.7 ± 0.71	2.70 ± 0.15
Diabetic Untreated	0.48 ± 0.14	21.0 ± 1.26	0.68 ± 0.07
Diab + Mg	1.03 ± 0.20#	23.1 ± 0.64	1.09 ± 0.32*#
Diab + Met	1.17 ± 0.24#	23.9 ± 0.33	0.98 ± 0.17*#
Diab + Mg + Met	0.98 ± 0.28#	23.6 ± 0.32	1.35 ± 0.28*#

***Values expressed as mean ± SEM;***^∗^ indicates values that are significantly different (p < 0.05) compared to control. ^#^ indicates values that are significantly different (p < 0.05) compared to diabetic untreated group.

### 3.3 Thyroid and renal function in control and experimental animals

Diabetic untreated animals exhibited a reduction in thyroid stimulating hormone (TSH) and triiodothyronine (T3) level compared to control and other experimental groups while thyroxine (T4) values obtained were not significantly different between control and all other experimental groups (Table 4). Assessment of renal function showed BUN, creatinine, total protein and albumin values that though increased in the diabetic untreated group were however not significantly different (P > 0.05) from control and other experimental groups (Table 5). Globulin levels though reduced in the diabetic untreated group were also not significantly different (P > 0.05) from values obtained in control and all other experimental groups (Table 5).

**Table 3:** Renal antioxidants and lipid peroxidation in normal and experimental animals.

	Reduced glutathione	Superoxide dismutase	Lipid peroxidation
Control	44.09 ± 2.00	1.33 ± 0.09	0.40 ± 0.07
Diabetic Untreated	40.49 ± 1.25	0.72 ± 0.03	0.73 ± 0.27
Diab + Mg	52.86 ± 4.95#	1.11 ± 0.13#	0.22 ± 0.06*#
Diab + Met	49.22 ± 5.71#	0.95 ± 0.12#	0.42 ± 0.17
Diab + Mg + Met	43.29 ± 1.69	1.07 ± 0.18	0.41 ± 0.07

***Values expressed as mean ± SEM;***^∗^ indicates values that are significantly different (p < 0.05) compared to control. ^#^ indicates values that are significantly different (p < 0.05) compared to diabetic untreated group.

**Table 5:** Kidney function tests in Control and Treated Animals.

	BUN	Creatinine	Total protein	Albumin	Globulin
Control	17.02 ± 0.20	0.58 ± 0.02	7.06 ± 0.27	3.26 ± 0.14	4.1 ± 0.17
Diabetic untreated	17.26 ± 0.17	0.62 ± 0.02	7.4 ± 0.03	3.28 ± 0.10	3.96 ± 0.21
Diab + Mg	16.48 ± 0.37	0.58 ± 0.02	7.04 ± 0.07	2.9 ± 0.16	4.06 ± 0.11
Diab + Met	16.82 ± 0.27	0.58 ± 0.02	7.04 ± 0.07	3.02 ± 0.09	4.2 ± 0.14
Diab + Mg + Met	16.4 ± 0.11	0.52 ± 0.02#	6.66 ± 0.15	2.76 ± 0.13	4.16 ± 0.14

***Values expressed as mean ± SEM.***^#^ indicates values that are significantly different (p < 0.05) compared to diabetic untreated group.

### 3.4 Histological evaluation of the kidney

**Plate 1:**
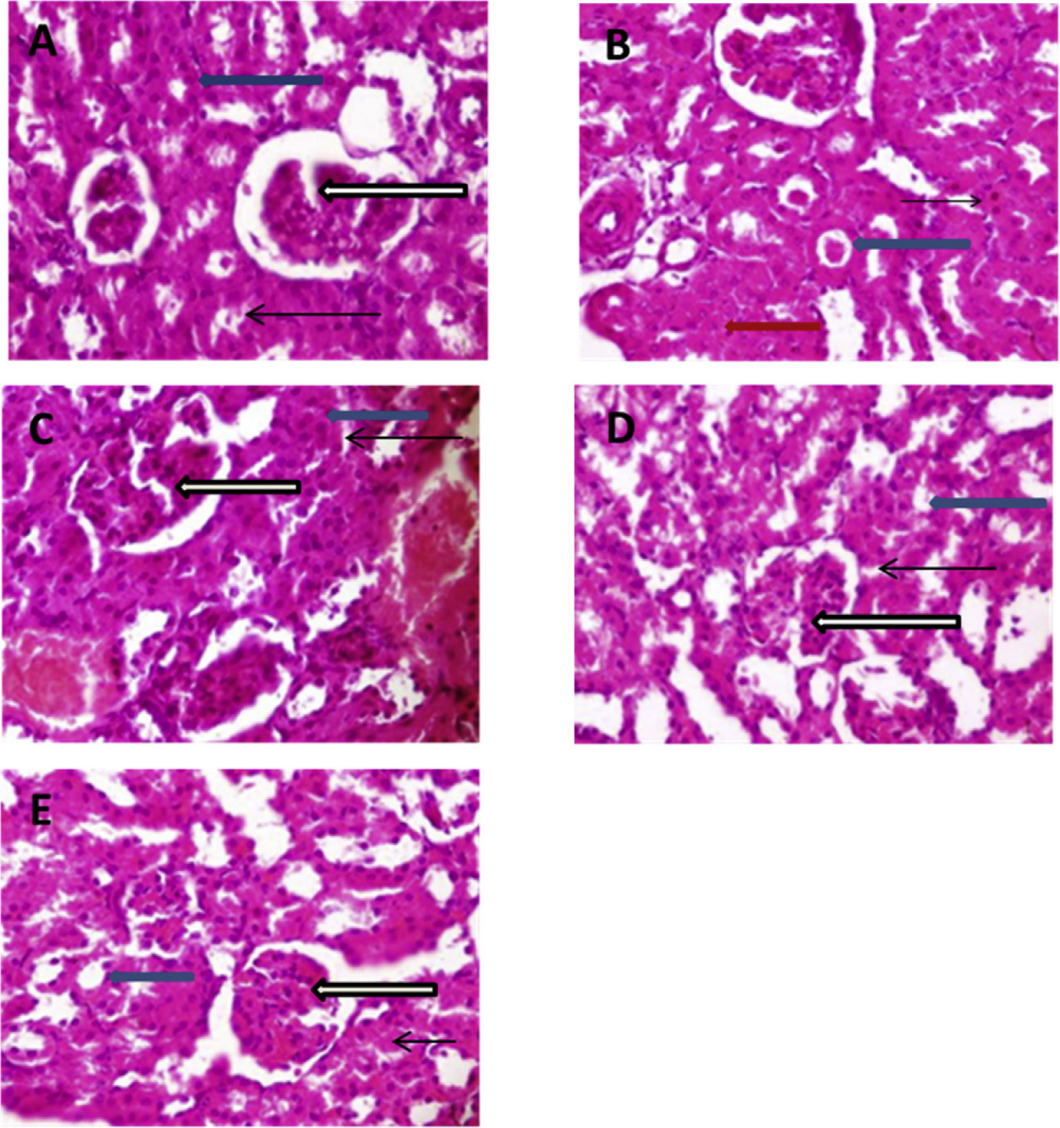
(A–E) Photomicrographs of Kidney sections (H and E stains) in control and experimental groups; a – Control, B – Diabetic untreated, C – Diabetic animal treated with Magnesium only, D – Diabetic animals treated with Metformin only, E – Diabetic animals treated with both Magnesium and Meformin.

Histological evaluation of the kidney for morphologic changes using H and E stains (Plate 1A–E) indicates that the diabetic untreated group (1B) had kidney samples showing poor architecture. The renal cortex in this group also showed some glomeruli with sclerosis (black arrow) and fused messangial cells, some renal tubules showing diffuse collapsed lumen (red arrow) and others showing the presence of eosinophilic renal cast within their lumen (blue arrow). Mild vascular congestion was also observed (white arrow) and the interstitial spaces seen appear limited (slender arrow) (1B). Kidney sample from control (1A), diabetic animals treated with metformin (1D) and diabetic animals co-treated with magnesium and metformin (1E) showed normal architecture with renal cortex showing normal glomeruli with normal messangial cells and capsular spaces (white arrow), the renal tubules including distal convoluted tubules and proximal convoluted tubules appear normal (blue arrow), the interstitial spaces appear normal (slender arrow). Furthermore, no pathological lesions were seen in these treatment groups. Diabetic animals treated with magnesium only (1C) had kidneys with poor architecture, however, the renal cortex in this group showed several normal glomeruli with normal messangial cells and capsular spaces (white arrow). Furthermore, the renal tubules observed demonstrate diffuse collapse of the lumen and luminal spaces are not seen (blue arrow); the interstitial spaces appear limited as the tubules appear compact (slender arrow). There is also mild to moderate vascular congestion noted (black arrow) (1C).

**Plate 2:**
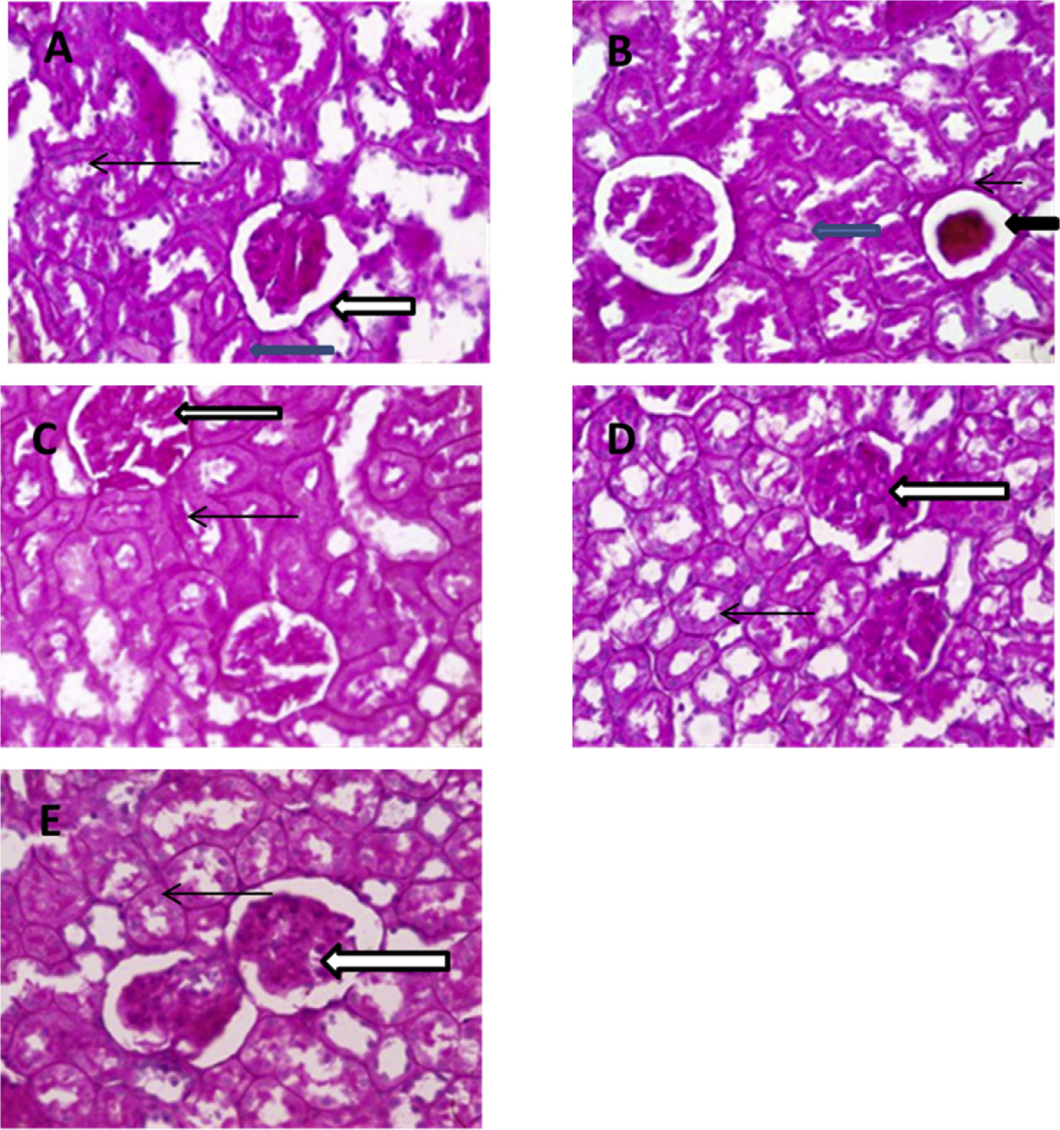
(A–E) Photomicrographs of Kidney sections (Periodic acid Schiff (PAS) stains) in control and experimental groups; a – Control, B – Diabetic untreated, C – Diabetic animal treated with Magnesium only, D – Diabetic animals treated with Metformin only, E – Diabetic animals treated with both Magnesium and Meformin.

Evaluation of the kidney samples using periodic acid Schiff stains for tubular aberrations (Plate 2A–E) showed that diabetic untreated group (2B) had poor architecture, their glomeruli show degrees of sclerosis and messangial cells in this group appear fused (black arrow). Few glomeruli appear void lacking apparatus (blue arrow), the basement membrane of the glomeruli appears thickened (slender arrow) and there is considerably loss of brush borders within the proximal convoluted tubules. Kidney samples in the control (2A) and experimental treatment groups (2C-E) indicate samples with normal basement membranes of the glomeruli (white arrow) and renal tubules (blue arrow). The messangial cells and the brush border of the proximal convoluted tubes were also PAS positive (slender arrow).

## 4 Discussion and conclusion

Despite recent advances in the management of diabetes mellitus, there still exists a high prevalence rate in the world population [19]. The increased morbidity and mortality associated with diabetes mellitus may be as a result of the multifaceted pathology of the syndrome, which results in it exerting its effects on various organ systems in the body [20]. According to Srinvansan et al. [14], and Binh *et al.* [21], the induction of experimental type-2-diabetes mellitus using high-fat diet and low-dose streptozotocin injection proceeds with increased body weight, increased insulin secretion and insulin resistance often resulting in hyperglycaemia. These manifestations were noted in the untreated diabetic group in this study and suggest that these animals had diabetes mellitus. Magnesium supplementation in this study, either alone or in combination with metformin, exerted a hypoglycemic effect, as did metformin in diabetic treated animals, which for magnesium may be ascribed to its documented potentiation of insulin secretory activities, its glucose regulatory and blood glucose stabilizing effects [7, 22]. Similarly the effects of Metformin, the most frequently prescribed first line therapy for individuals with type 2 diabetes [23], seen in this study may be linked to its ability to increase insulin mediated glucose utilization in peripheral tissues (such as muscle and liver) and thus improve glycemic control [24].

Prolonged hyperglycemia as observed in diabetes mellitus has been reported to exert deleterious effects via various mechanisms some of which include the polyol pathway, activation of the diacylglycerol (DAG)/protein kinase C (PKC) pathway, increased oxidative stress, increased advanced glycation end products (AGE) formation and action, and increased hexosamine pathway [25]. A prevalence of altered thyroid status has been observed in diabetes mellitus [6] with little or no information on the precise mechanism of its occurrence. It is however known that in diabetic patients, the nocturnal TSH peak is blunted or abolished, TSH response to thyroid releasing hormone (TRH) is impaired and T3 levels are reduced [6].

This study suggests the presence of hypothyroidism in the diabetic untreated animals as TSH and T3 values seen were reduced compared to control and other experimental groups, which is in accordance with Baydas *et al* [24] and may partially be ascribed to an impaired peripheral conversion of T4 to T3 resulting from decreased activity of type 1 liver monodeiodinase (D1) [26,27] that has been reported in diabetic conditions [28]. The reduction in TSH level in the untreated diabetic animals is also consistent with the report of Pasupathi *et al* [29] and may be ascribed to impairment in the negative feedback control of TSH secretion by T3. Oral magnesium supplementation, either alone or in combination with metformin appeared to correct the TSH and T3 reductions seen in the diabetic untreated group and this may be attributed to either an improvement in glycemic control [28] following magnesium and metformin administration or a potentiation of glutathione activity by magnesium [30], which has been reported to facilitate improved conversion of T4 to T3 [24]. However, thyroxine level across groups was not changed. This is consistent with Donckier [31] who also observed near- normal serum T4 level despite poor glycemic control.

In hypothyroidism, it has been reported that there is usually a reduction in renal blood flow (RBF) and glomerular filtration rate (GFR) arising from a reduction in cardiac output, increased peripheral vascular resistance, reduced renal response to vasdilators and a reduced expression of renal vasodilators [5]. Furthermore, pathologic changes in the glomerular structure such as glomerular membrane thickening and messangial matrix expansion [21] have also been reported to contribute to a reduced RBF and GFR in hypothyroidism. This study shows a histological profile in the diabetic untreated rats that is consistent with the manifestations of hypothyroidism on renal structures that can lead to a reduction in GFR. This suggests that aside from the direct effects of diabetes on the kidneys, hypothyroidism that accompanies diabetes mellitus may also contribute to renal pathologies seen in diabetes. Furthermore, oxidative stress was observed in the renal tissue as renal antioxidants assessed were depleted and accompanied by an increase in lipid peroxidation. This is consistent with other studies [32, 33] and suggests impairment in renal antioxidant balance and likely renal impairment.

Magnesium has been reported to act as an antioxidant, be a precursor molecule for glutathione as well as potentiate glutathione production in experimental diabetic animals [30]. Glutathione has been described as a power intracellular antioxidant and detoxifier hence its potentiation by magnesium may partly be responsible for the improved renal histology and antioxidant status in the magnesium and metformin [34] treated diabetic groups respectively. Furthermore the combination of normoglycemia and amelioration of thyroid dysfunction after magnesium or metformin treatment might also account for the observed alleviation of renal oxidative stress seen in the treated diabetic groups.

Quantitative assessment of renal function in the diabetic untreated group via evaluation of blood urea nitrogen, creatinine, total protein, albumin and globulin levels [35] show slight elevations in renal function indices particularly for creatinine which was elevated in the diabetic untreated compared to diabetic animals co-treated with magnesium and metformin. This suggests impaired kidney function or kidney disease in the diabetic untreated group. It is speculated that had duration of this study had been increased, clear quantitative renal function differences would be more apparent.

This study however has few limitations; first basal and final magnesium status was not ascertained in this study, as magnesium deficiency is a proposed factor in the pathogenesis and progression of diabetic complications. Hypomagnesaemia has also been reported in diabetics. Evaluation of final magnesium status may have given information as to whether magnesium status was restored after supplementation or not. Secondly glomerular filtration rate has been described as the gold standard for measuring renal function. Its measurement in this study would have given further credence to the histological finding within the renal tissue that suggests that GFR would have been altered in the diabetic untreated animals. In subsequent studies on renal function, this would be taken into consideration and factored into the experimental procedures.

In conclusion, this study suggests that in uncontrolled experimental type-2-diabetes there is impairment in thyroid function leading to reductions in serum thyroid stimulating hormone and triiodothyronine but not thyroxine levels. The observed thyroid dysfunctions may contribute to renal impairment that usually accompanies experimental type-2-diabetes. The study also suggests that treatment with oral magnesium may cause a partial restoration of thyroid function that may impede the development of renal dysfunction in experimental type-2-diabetic rats.

## Declarations

### Author contribution statement

Abayomi Ige: Conceived and designed the experiments; Performed the experiments; Analyzed and interpreted the data; Contributed reagents, materials, analysis tools or data; Wrote the paper.

Rachel Chidi, Evelyn Egbeluya, Rofiat Olajumoke Jubreel: Performed the experiments; Analyzed and interpreted the data; Contributed reagents, materials, analysis tools or data.

Ben Adele: Performed the experiments; Analyzed and interpreted the data; Wrote the paper.

Elsie Adewoye: Analyzed and interpreted the data; Contributed reagents, materials, analysis tools or data; Wrote the paper.

### Funding statement

This research did not receive any specific grant from funding agencies in the public, commercial, or not-for-profit sectors.

### Competing Interest Statement

The authors declare no conflict of interest.

### Additional Information

No additional information is available for this paper.
